# Development of Nanobodies against Mal de Río Cuarto virus major viroplasm protein P9-1 for diagnostic sandwich ELISA and immunodetection

**DOI:** 10.1038/s41598-021-99275-z

**Published:** 2021-10-08

**Authors:** Gabriela Llauger, Demián Monti, Matías Adúriz, Ema Romão, Analía Delina Dumón, María Fernanda Mattio, Andrés Wigdorovitz, Serge Muyldermans, Cécile Vincke, Viviana Parreño, Mariana del Vas

**Affiliations:** 1 Instituto de Agrobiotecnología y Biología Molecular (IABIMO), CICVyA INTA, UEDD INTA/CONICET, Buenos Aires, Argentina; 2 INCUINTA, Instituto de Virología e Innovaciones Tecnológicas (IVIT), CICVyA INTA, UEDD INTA/CONICET, Buenos Aires, Argentina; 3 Lab of Cellular and Molecular Immunology, Vrije Universiteit Brussel, Brussels, Belgium; 4 Consejo Nacional de Investigaciones Científicas y Técnicas (CONICET), Unidad de Fitopatología y Modelización Agrícola (UFYMA), Córdoba, Argentina; 5 Instituto Nacional de Tecnología Agropecuaria (INTA), Centro de Investigaciones Agropecuarias (CIAP), Instituto de Patología Vegetal (IPAVE), Córdoba, Argentina; 6 Liaoning Key Laboratory of Molecular Recognition and Imaging, School of Bioengineering, Dalian University of Technology, Dalian, People’s Republic of China; 7 Myeloid Cell Immunology Lab, VIB Center for Inflammation Research, Brussels, Belgium

**Keywords:** Antibody generation, Antibody isolation and purification, ELISA, Immunoblotting, Immunohistochemistry, Immunoprecipitation, Infectious-disease diagnostics

## Abstract

Mal de Río Cuarto virus (MRCV) is a member of the genus *Fijivirus* of the family *Reoviridae* that causes a devastating disease in maize and is persistently and propagatively transmitted by planthopper vectors. Virus replication and assembly occur within viroplasms formed by viral and host proteins. This work describes the isolation and characterization of llama-derived Nanobodies (Nbs) recognizing the major viral viroplasm component, P9-1. Specific Nbs were selected against recombinant P9-1, with affinities in the nanomolar range as measured by surface plasmon resonance. Three selected Nbs were fused to alkaline phosphatase and eGFP to develop a sandwich ELISA test which showed a high diagnostic sensitivity (99.12%, 95% CI 95.21–99.98) and specificity (100%, 95% CI 96.31–100) and a detection limit of 0.236 ng/ml. Interestingly, these Nanobodies recognized different P9-1 conformations and were successfully employed to detect P9-1 in pull-down assays of infected maize extracts. Finally, we demonstrated that fusions of the Nbs to eGFP and RFP allowed the immunodetection of virus present in phloem cells of leaf thin sections. The Nbs developed in this work will aid the study of MRCV epidemiology, assist maize breeding programs, and be valuable tools to boost fundamental research on viroplasm structure and maturation.

## Introduction

Cereal crops including maize, rice and wheat are the most important sources of calories and nutrition for the human population and are essential for livestock feed. Yields are limited by a number of factors including abiotic and biotic stresses and are challenged by climate change. Mal de Río Cuarto is a severe maize disease that causes significant economic losses in Argentina^1^, one of the most important corn exporters worldwide. The disease is caused by Mal de Río Cuarto virus (MRCV), a member of the genus *Fijivirus* within the family *Reoviridae* that is transmitted in a persistent and propagative manner by delphacid planthoppers^2,3^. Other members of the genus cause important maize and rice diseases in Eastern Asia^4,5^ and in Mediterranean countries^6–8^. Their genome is composed of ten dsRNA segments that are tightly packed into icosahedric double-layered capsids, and code for at least 13 proteins. Within the *Reoviridae* family, virus replication and assembly occur in highly organized and dynamic cytoplasmic structures called viroplasms or viral factories that are composed of viral and host proteins as well as viral RNA^9^. The assembly of the viroplasm is an early and crucial step during infection: impairment of the expression of major viroplasm proteins leads to immunity in transgenic plants^10^ and animal cells^11–14^. MRCV viroplasms are basically composed of non-structural viral proteins P9-1 and P6. The P9-1 is the major viroplasm component, has RNA binding ability, ATPase activity and multimerizes into homo-oligomers^15^, whereas P6 is a minor component^16^. P9-1 and P6 self-interact, interact with each other, and contain PEST motifs for putative proteasome-mediated degradation^17^.

In plants, MRCV infection is phloem-limited and causes hormone imbalance and sugar accumulation in leaves^18^. The severity of symptoms is directly associated with yield loss^19^ and depends on maize genotype^20^, winter environmental variables such as temperature and rainfall that affect insect vector populations^21^, as well as on the phenological stage of the plants at the time of infection. When infection takes place at early stages of development, maize plants show severe symptoms including general stunting, shortening of internodes, increased tillering, defective grain production and discrete tumour proliferations along the veins^22^. Late infection leads to milder symptoms. Occasionally, coinfection with other virus species can mask MRCV typical symptoms^23,24^. Studies on MRCV epidemiology including virus cellular and subcellular distribution in natural infections of plants and insect vectors are comparatively scanty, mainly because of the lack of adequate tools to monitor the virus infection.

Camelids have a unique immune system producing a particular class of antibodies devoid of light chains called heavy-chain-only antibodies (HCAbs)^25^. Llamas (*Lama glama*) are domesticated South American camelids widely distributed throughout the Andes^26^, and express about 20–40% of HCAbs in their sera^25^. Nanobodies (Nbs) are recombinant, single-domain fragments derived from the variable domains (VHHs) of the camelid HCAbs, and constitute the smallest proteins with antigen binding capacity^27^. Nbs have dimensions in single digit nanometer scale and present a number of properties that make them an ideal tool for many research, diagnostic, therapeutic and industrial applications. These beneficial properties include the high affinity and specificity for their targets, high stability and solubility, nanoscale size and superior accessibility to cryptic cleft regions, deep tissue penetration and highly efficient expression in bacterial hosts^28^. The Nb tailoring and production is simple and extraordinarily versatile. In particular, for diagnostic purposes, the fusion of Nbs to molecules such as alkaline phosphatase, horseradish peroxidase or biotin is easily achieved using molecular cloning techniques^29–32^.

In this work, we generated, selected and characterized Nbs with nanomolar affinity to P9-1. These Nbs were used to develop a highly sensitive sandwich ELISA that specifically and rapidly detected MRCV infection in plants. Furthermore, we probed these Nbs against two variants of P9-1 and results suggest that each Nb interacts with its target in a distinctive manner. In addition, we showed that these Nbs could be employed in pull-down assays of P9-1 in infected leaf extracts and, when fused to eGFP or RFP, to immunolocalize the virus in phloem tissue of infected plant leaves. Overall, these Nbs are promising tools for diagnostic purposes and to decipher the interaction of MRCV with plant and insect hosts.

## Results

### Identification of high affinity MRCV P9-1 specific Nanobodies

To obtain Nanobodies specific against P9-1, a llama was immunised with a recombinant version of the major MRCV viroplasm protein P9-1. The llama developed a strong antibody response to the viral protein after four doses. Fifteen days after the last immunisation, peripheral blood cells from the llama were collected and employed to construct a VHH library in pMECS, containing 3.3 × 10^7^ transformants. After three rounds of phage-display selection of Nbs against immobilized P9-1 recombinant protein, 75 clones (out of 94 clones tested) yielded a positive signal on a phage-ELISA. These steps are summarized in Supplementary Fig. S1. Of those, 40 clones yielding the higher signals were sequenced, resulting in 27 clones of unique sequence aligned according to the International Immunogenetics Information System (IMGT)^33^ and grouped into 12 clonally related families, based on their CDR3 sequences (data not shown). Eight Nbs from six different families were selected for their high expression levels in *E. coli* WK6 periplasm, and purified by immobilized metal affinity chromatography (IMAC) followed by Size Exclusion Chromatography (SEC) (Supplementary Fig. S2).

To characterize the molecular interactions of the eight selected Nbs with P9-1, the binding kinetics were determined by surface plasmon resonance (SPR). All eight Nbs gave at their maximum loading a similar maximum response unit (RU_max_) value (around 140–160) in agreement with a 1:1 binding stoichiometry of Nb over P9-1 monomer, that should therefore be used for affinity calculations. Sensorgrams and kinetic constants are shown in Supplementary Fig. S3 and Table 1, respectively. Three Nanobodies, namely Nb1, Nb25 and Nb13 belonging to clonally individual families, presented the highest binding affinities, with equilibrium dissociation constants (K_D_) ranging between 3.05 and 71.61 nM. These Nbs were selected to assess their performance in diagnosis and immunodetection.

**Table 1:** Kinetic constants of the selected Nbs.

Nanobody	k_a_ (1/Ms)	k_d_ (1/s)	K_D_ (nM)
Nb 1	3.805 × 10^6^	0.01160	3.05
Nb 13	7.896 × 10^5^	0.05655	71.61
Nb 25	4.283 × 10^5^	0.00790	18.44
Nb 30	3.774 × 10^5^	0.08012	212.3
Nb 36	3.774 × 10^6^	0.16800	136.1
Nb 85	2.550 × 10^6^	0.28380	111.3
Nb 89	1.114 × 10^5^	0.02748	246.8
Nb 92	5.901 × 10^5^	0.06280	106.4

Kinetic rate constants of association (k_a_), and dissociation (k_d_), and the corresponding equilibrium dissociation constants (K_D_) of the selected Nanobodies against MRCV P9-1 viroplasm protein as determined by SPR.

### Development of capture and detection Nb-based tools and selection of the best Nb pair for MRCV diagnosis

To obtain the most efficient Nanobody-based sandwich ELISA detecting MRCV presence in infected leaf samples, we searched for the optimal P9-1 capturing and detection Nb pair. Considering that a proper orientation of the capturing Nb accounts for a lower limit of detection^31^, fusions of the Nbs to eGFP were constructed. The larger size and globular structure of eGFP (26.9 kDa) is more likely to attach to the plate, thereby favouring the accessibility and orientation of the Nb to capture its antigen^31^. All three fusions were expressed in *E. coli* SHuffle strain and purified from soluble cytoplasmic extracts rendering high amounts of bright green recombinant Nb fusion proteins.

To avoid inefficient chemical conjugation of enzymes to Nbs, alkaline phosphatase fusions to the three Nbs were generated (Nb1:AP, Nb13:AP and Nb25:AP), expressed in *E. coli* BL21 strain and purified from periplasmic extracts for its use in antigen detection.

Fijivirus-infected plants were occasionally reported as asymptomatic^34,35^. Therefore, the presence of virus in all the plant samples that were collected and initially grouped by visual observation into symptomatic or asymptomatic, were individually confirmed by RT-PCR, before evaluating the performance of our Nbs-based tools on these samples. According to this method, 16% of the asymptomatic and 100% of the symptomatic plants were indeed infected (Supplementary Fig. S4). Then, pools of ten MRCV-infected or non-infected plants were built and used for the following experiments.

**Figure 1:**
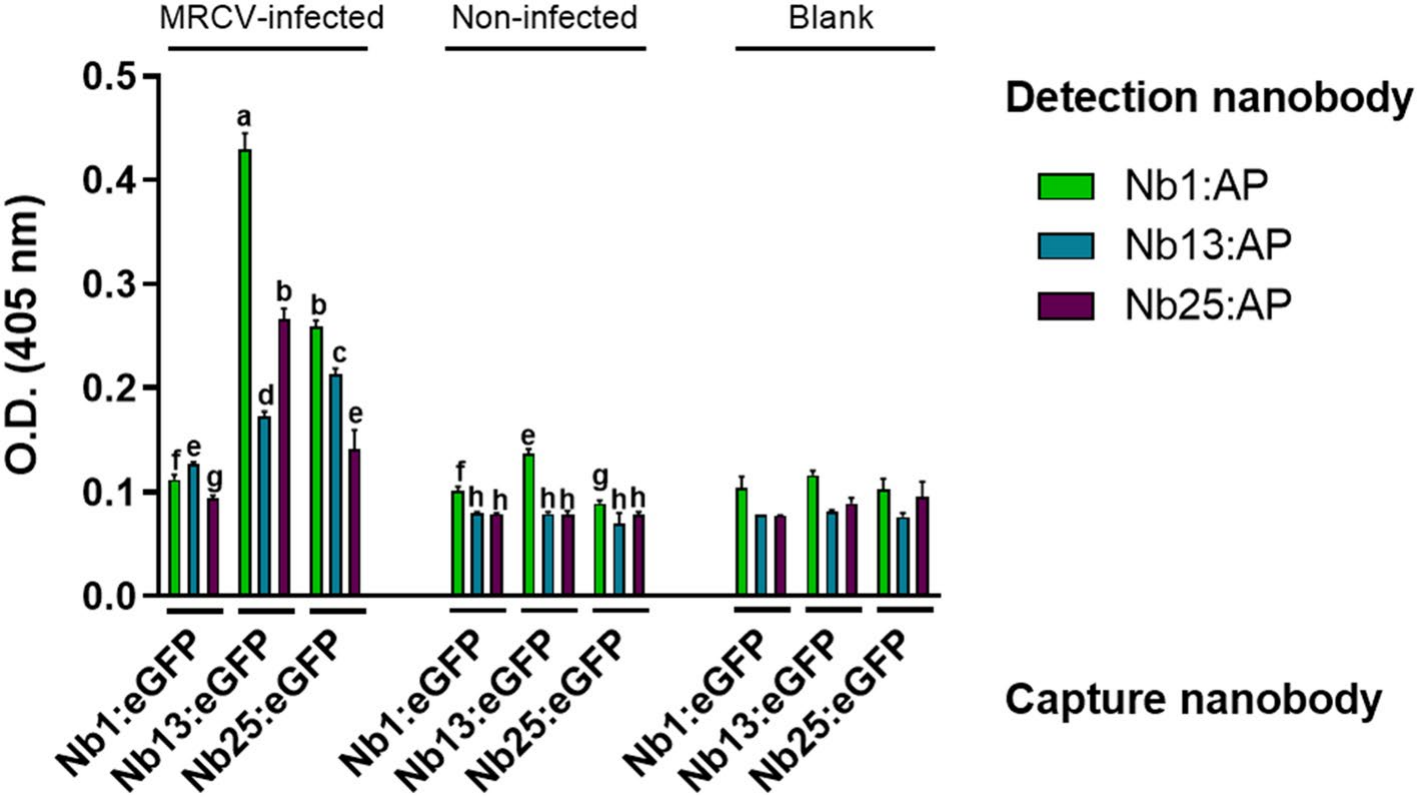
Definition of the best capturing-detection Nb combination for sandwich ELISA. Three different Nbs were independently used as capturing Nb fused to eGFP (Nb:eGFP), and as detecting Nb fused to alkaline phosphatase (Nb:AP), in all possible combinations. Wells were coated with 100 µl of 5 ng/µl of Nb:eGFP, incubated with 100 µl of 0.5 ng/µl of Nb:AP and detected with 100 µl of 1 mg/ml of pNPP. ANOVA followed by Tukey’s multiple comparison test was performed. Bars denoted by the same letter are not statistically significant (p > 0.05).

Next, the three Nb:eGFP fusions were evaluated for their abilities to efficiently capture P9-1 in the naturally MRCV-infected maize pool protein extracts, while the Nb:AP fusions were employed for detection. All nine possible combinations of capture and detection Nbs were tested. As shown in Fig. 1, both Nb13:eGFP and Nb25:eGFP were able to capture P9-1 in MRCV-infected plant pools. Interestingly, Nb1:eGFP displayed the worst performance as a capture Nb in spite of having the highest affinity to P9-1. None of the Nb:eGFP/Nb:AP combinations gave rise to positive signal in non-infected plant extracts when compared to blank measurements. Furthermore, when any given Nanobody was used both for capture and detection, the signal was low, most likely because of epitope competition. Overall, the most sensitive pair for P9-1 detection was Nb13:eGFP/Nb1:AP (Fig. 1). Importantly, these results indicate that the Nbs raised and selected against recombinant P9-1 were also able to recognize *wild-type* P9-1 present in naturally occurring MRCV infections in maize.

### Optimization of a nanobody-based sandwich ELISA protocol

**Figure 2:**
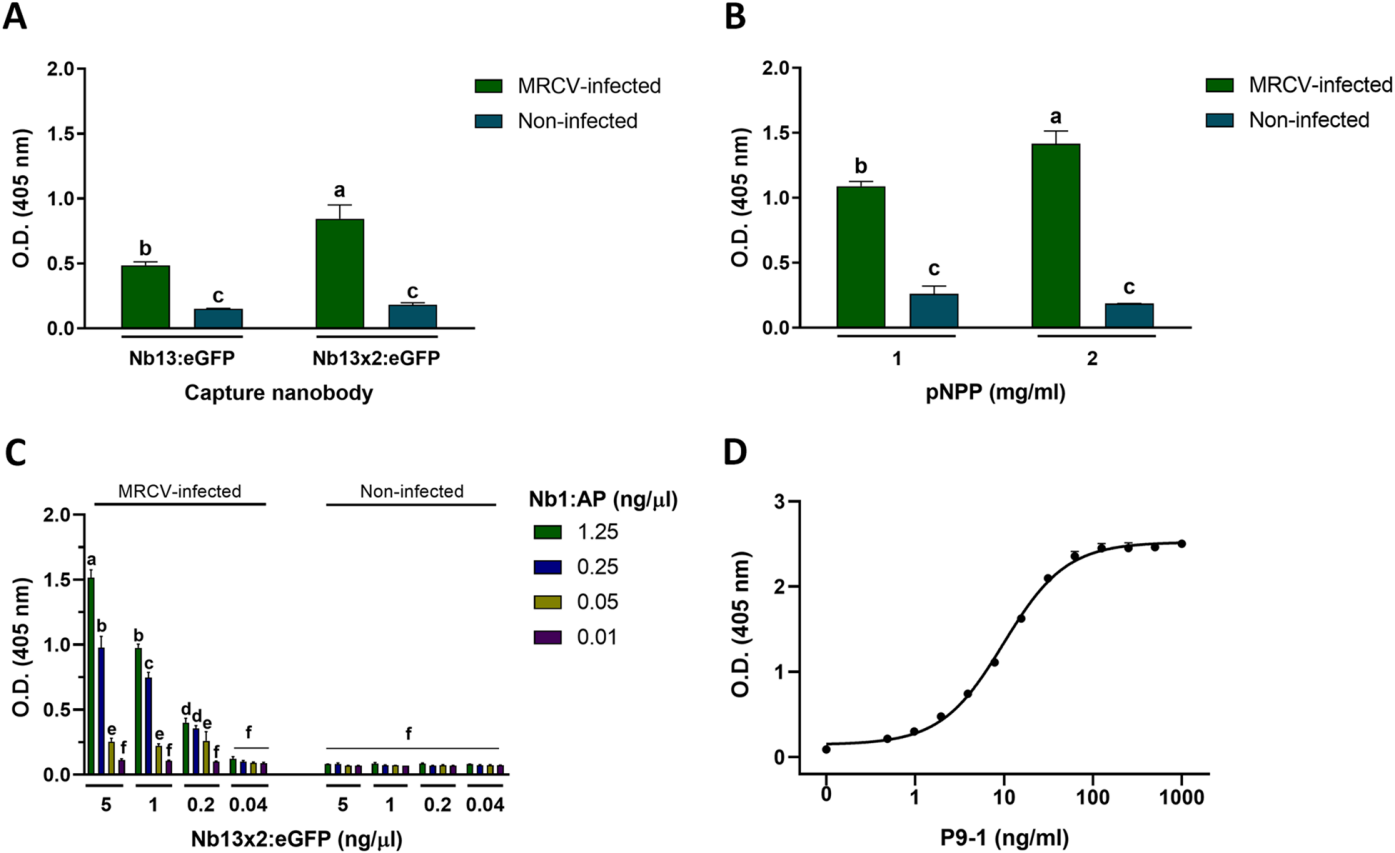
Sandwich ELISA optimization. (**A**) Assessment of bivalent Nb13 (Nb13 × 2:eGFP) as capturing antibody. Coating was performed with 100 µl of 6.83 ng/µl to achieve an equimolar amount of monovalent and bivalent Nb. (**B**) Assessment of different pNPP concentrations. (**C**) Checkerboard titration of Nb13 × 2:eGFP and Nb1:AP. (**D**) Four-parameter logistic model fit for the determination of the limit of detection (LOD). Values are means ± SD (n = 3). ANOVA followed by Tukey’s multiple comparison test was performed. Bars denoted by the same letter are not statistically significant (p > 0.05).

Since it is well established that bivalent antibodies improve the assay sensitivity^36^, we constructed and purified a bivalent version of Nb13 fused to eGFP (Nb13 × 2:eGFP). As shown in Fig. 2A, the capturing bivalent Nb13 × 2:eGFP yielded a  twofold signal increase in ELISA, compared to the monovalent Nb13:eGFP. Also, we assayed two different p-Nitrophenyl Phosphate (pNPP) concentrations (Fig. 2B), and a 35% signal increase was obtained with 2 mg/ml instead of 1 mg/ml pNPP. Sandwich ELISA protocol was slightly optimized by coating with Nb13 × 2:eGFP 4 h at 37 °C instead of overnight (ON) at 4 °C, extending the incubation period of the plant extracts with the coated plates from 1 h at 37 °C to ON at 4 °C and by diluting Nb1:AP in plant extraction buffer instead of PBS. These changes gave rise to higher OD_405nm_ for infected plants and lower background signals. Next, we determined the optimal concentrations of Nb13 × 2:eGFP and Nb1:AP in a checkerboard titration. The best performance was obtained using 5 ng/µl of Nb13 × 2:eGFP and 1.25 ng/µl of Nb1:AP (Fig. 2C). Finally, we established that the limit of detection (LoD) of purified recombinant P9-1 spiked in non-infected plant extracts (Fig. 2D). The data was fitted to a 4-parameter logistic model and the LoD was determined to be 0.236 ng/ml.

### Evaluation of the Nb-based sandwich ELISA specificity and sensitivity

**Figure 3:**
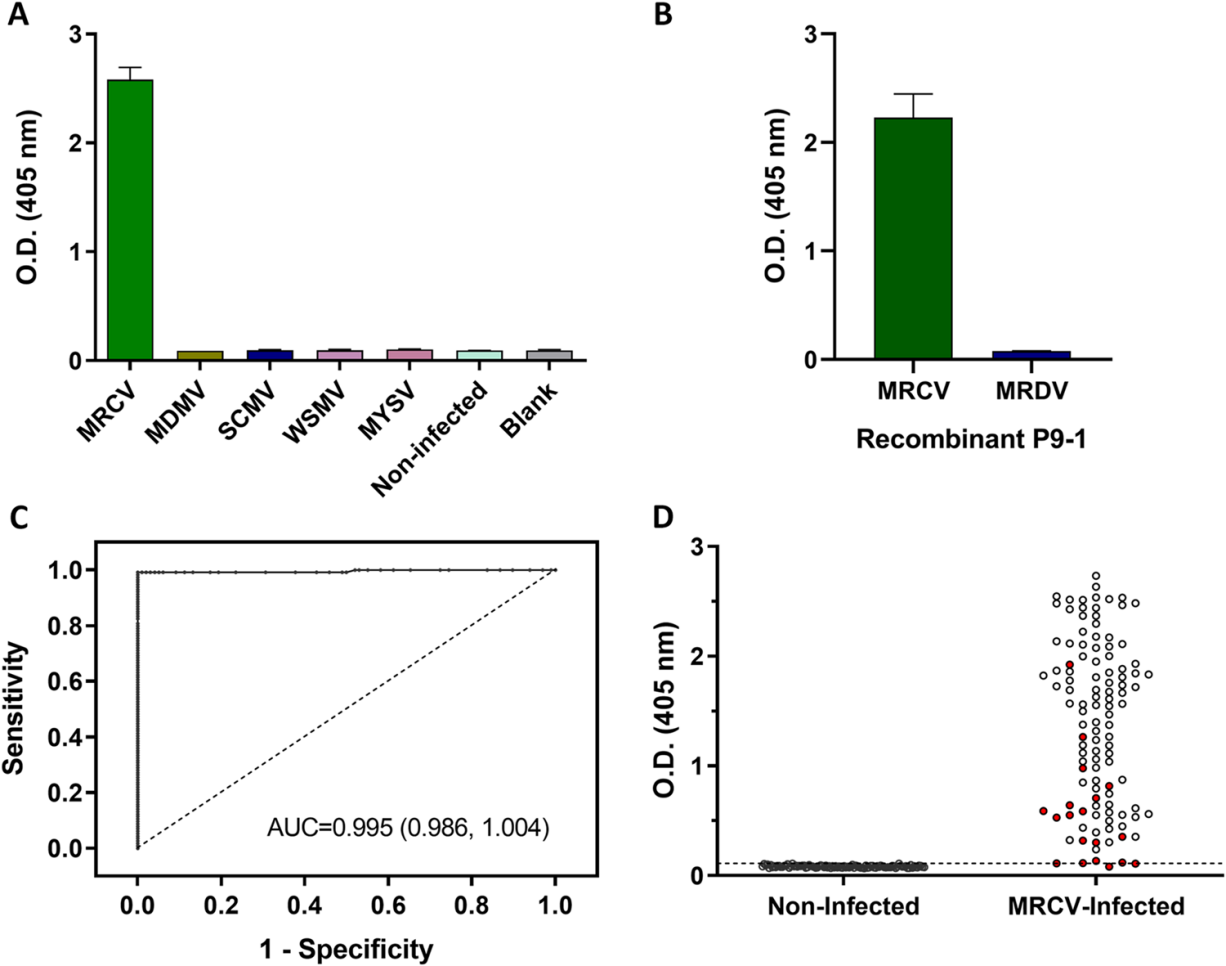
Specificity and sensitivity assessment of Nb-based sandwich ELISA. (**A**) For the analysis of the specificity, four different maize-infecting viruses were evaluated, MRCV was used as a positive control. Maize dwarf mosaic virus (MDMV, *Potyvirus*, *Potyviridae*), Sugarcane mosaic virus (SCMV, *Potyvirus*, *Potyviridae*), Wheat streaked mosaic virus (WSMV, *Tritimovirus*, *Potyviridae*), Maize yellow striate virus (MYSV, *Cytorhabdovirus*, *Rhabdoviridae*); (**B**) Sandwich ELISA on recombinant MRCV P9-1 and MRDV P9-1. Values are means ± SD (n = 3) (**C**) Receiver-Operating Characteristic (ROC) analysis from a total of 98 non-infected and 114 MRCV-infected samples. (**D**) Dot diagram for the 212 samples evaluated. Dashed line represents the cut-off obtained from the ROC curve analysis. Red dots indicate the infected plants classified as asymptomatic whereas white dots indicate those classified as symptomatic.

To establish the analytical specificity of the Nb-based MRCV sandwich ELISA we included in our test four gramineae viruses frequently infecting maize in Argentina: Maize dwarf mosaic virus (MDMV, *Potyvirus*, *Potyviridae*), Sugarcane mosaic virus (SCMV, *Potyvirus*, *Potyviridae*), Wheat streaked mosaic virus (WSMV, *Tritimovirus*, *Potyviridae*) and the proposed new Cytorhabdovirus Maize yellow striate virus (MYSV, *Rhabdoviridae*)^37^. MRCV-infected leaf plant samples were used as a positive control. As shown in Fig. 3A the Nanobody-based sandwich ELISA developed in this work did not cross-react with any of the other gramineae viruses. ELISA specificity was further evaluated by assessing possible cross-reaction to fijivirus Maize rough dwarf virus (MRDV) P9-1. Both proteins have a 62.1% amino acid sequence identity^38^. In doing so, non-infected plant extracts were spiked with purified recombinant MRDV P9-1. MRCV P9-1 was included as positive control. As shown in Fig. 3B, the ELISA test was highly specific.

Finally, the ELISA cut-off value was determined using 114 MRCV-infected and 98 non-infected samples, as determined by RT-PCR. The ELISA accuracy was evaluated and a Receiver-Operating Characteristic (ROC) curve was constructed (Fig. 3C). The area under the ROC curve (AUC) was 0.995 (95% CI 0.986–1.004) demonstrating that the ELISA is highly predictable. Furthermore, cut-off was set at an O.D. of 0.110 resulting in a sensitivity of 99.12% (95% CI 95.21–99.98) and a specificity of 100% (95% CI 96.31–100), meaning that most samples were correctly classified. Figure 3D shows the distribution of the absorbance values of MRCV-infected and non-infected samples. Average absorbance of symptomatic infected samples was 1.564 ± 0.708 while it was 0.538 ± 0.471 for asymptomatic infected samples.

### Identification of differential epitope recognition sites on P9-1 and P9-1 ΔC-arm

**Figure 4:**
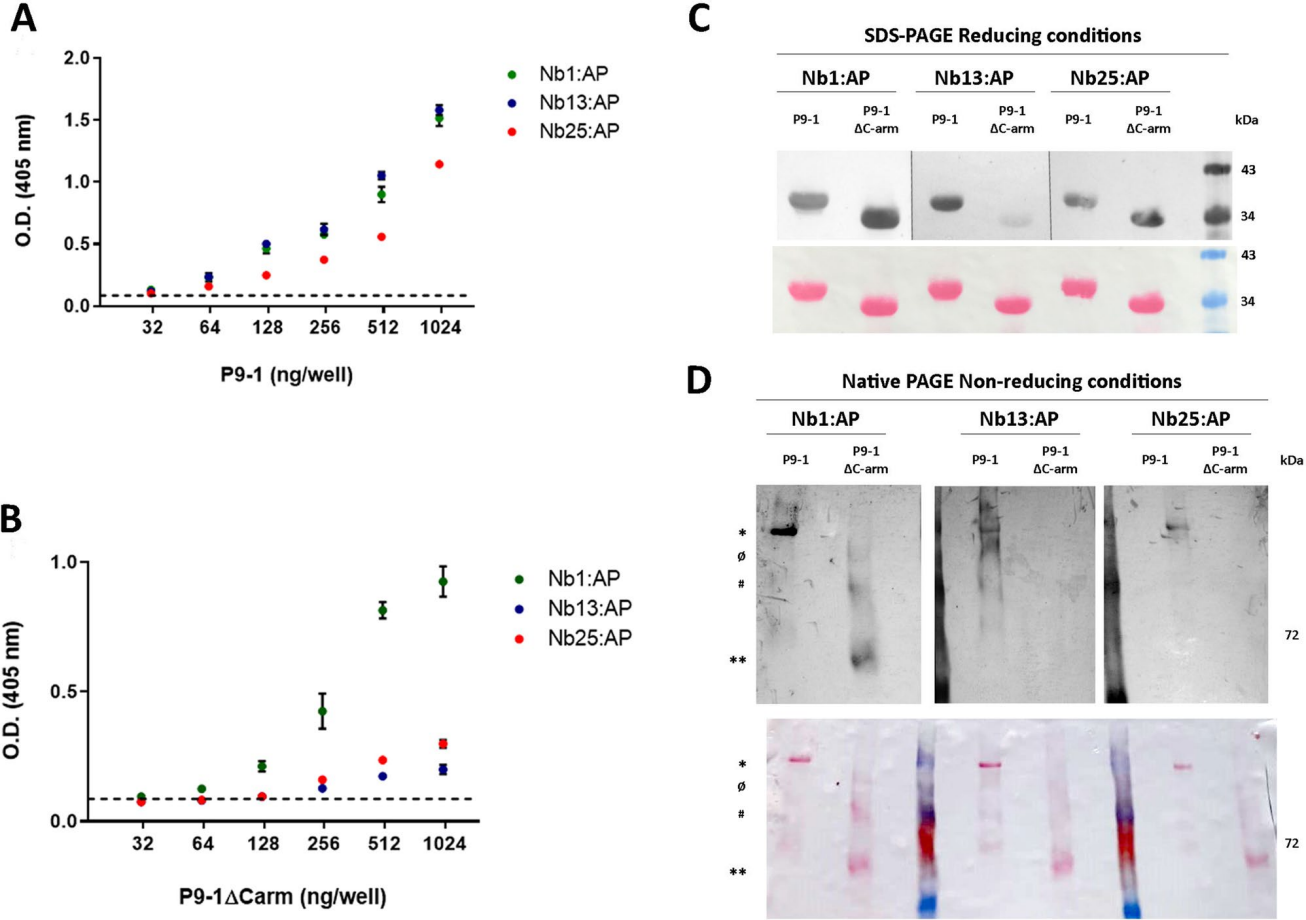
P9-1 and P9-1 ΔC-arm detection by western blot and direct ELISA with Nb:AP fusions. For direct ELISAs, plates were coated with either twofold dilutions of P9-1 (**A**) or P9-1 ∆C-arm (**B**) from 1000 to 31.25 ng/well and Nb:AP fusions were added in 1.25 ng/µl to determine their specificity towards *wild type* P9-1 and P9-1 ∆C-arm. Values are means ± SD (n = 3, experimental triplicates). Dashed lines indicate assay cut-off (mean of blanks plus 3*SD of blanks). (**C**) 10 µg of purified P9-1 and P9-1 ΔC-arm were subjected to SDS-PAGE. In reducing conditions, both proteins migrate as monomers. (**D**) 5 µg of purified P9-1 and P9-1 ΔC-arm were subjected to a native-PAGE. In these non-reducing conditions, P9-1 (indicated with *) migrates as a homomultimer, while multimerization of P9-1 ΔC-arm (**) is affected. The presence of P9-1 ΔC-arm aggregates can be detected as faint bands of higher molecular weights (ø and #). In (**C**) and (**D**), detection was performed with the different Nbs fused to alkaline phosphatase (AP) and by adding NBT/BCIP. Total protein load and transfer is revealed by Ponceau S staining below each western blot. In both cases, PageRuler™ prestained ladder was employed as protein molecular marker. However, since it is a size standard for SDS-PAGE, only the red standard protein of 72 kDa could be identified in the native-PAGE, serving only as an approximate reference in the latter case. Blots from (**C**) and (**D**) were cropped and grouped to simplify the Figure**.**

Crystallographic analysis of the fijivirus Rice black streaked dwarf virus (RBSDV) revealed that P9-1 forms octamers. Four pairs of dimers are held together by carboxy-terminal regions (24 residues) resembling arms (C-arms), giving rise to cylindrical octamers with an internal pore. Furthermore, the removal of the C-arms gives rise to dimers and disrupts the formation of viroplasm-like structures (VLSs) in vivo^39^. Accordingly, C-arms are required for P9-1 self-interactions in yeast two-hybrid analysis^17^. Considering P9-1 C-arm contribution to viroplasm formation, we performed a direct ELISA to assess whether Nb1, Nb13 and Nb25 were able to distinguish between P9-1 and a mutant version lacking the C-arm (P9-1 ΔC-arm). It was observed that all of the Nbs bound recombinant P9-1 and gave a positive signal even at 31.25 ng/well, the lowest protein concentration tested. The best response was observed for Nb13:AP, even when Nb13 presented the highest K_D_ value (Fig. 4A). Remarkably, only Nb1:AP gave a positive signal against P9-1 ΔC-arm at concentrations of 31.25 and 62.5 ng/well (Fig. 4B). Although Nb13:AP and Nb25:AP reacted against P9-1 ∆C-arm at higher antigen concentrations, signal was considerably lower than Nb1:AP. To deepen into this observation, western blot analyses were performed on P9-1 and P9-1 ΔC-arm separated by denaturing or native PAGE and directly probed with Nb1:AP, Nb13:AP or Nb25:AP. All three Nb:APs detected both multimeric (native) and monomeric (reduced) forms of P9-1 (Fig. 4C,D), suggesting that the epitopes being recognized are present in both P9-1 conformations. In turn, Nb1:AP recognized P9-1 ΔC-arm exposed to reducing and denaturing conditions (Fig. 4C), and also the native, non-reduced form (Fig. 4D), indicating that deletion of the C-arm does not impair Nb1:AP epitope recognition. In contrast, for Nb13 only a faint signal was observed against monomeric P9-1 ΔC-arm in the reducing condition, suggesting that this Nb may target either the C-arm or an epitope present in P9-1 and absent in P9-1 ΔC-arm final conformation. Finally, Nb25 only associated with P9-1 ΔC-arm in its reduced form. Taken together, our results indicate that the three Nbs recognize distinct epitopes on P9-1, mostly evidenced by their differential detection of the mutant P9-1 ΔC-arm conformers.

### P9-1 specific Nanobodies as research tools

**Figure 5:**
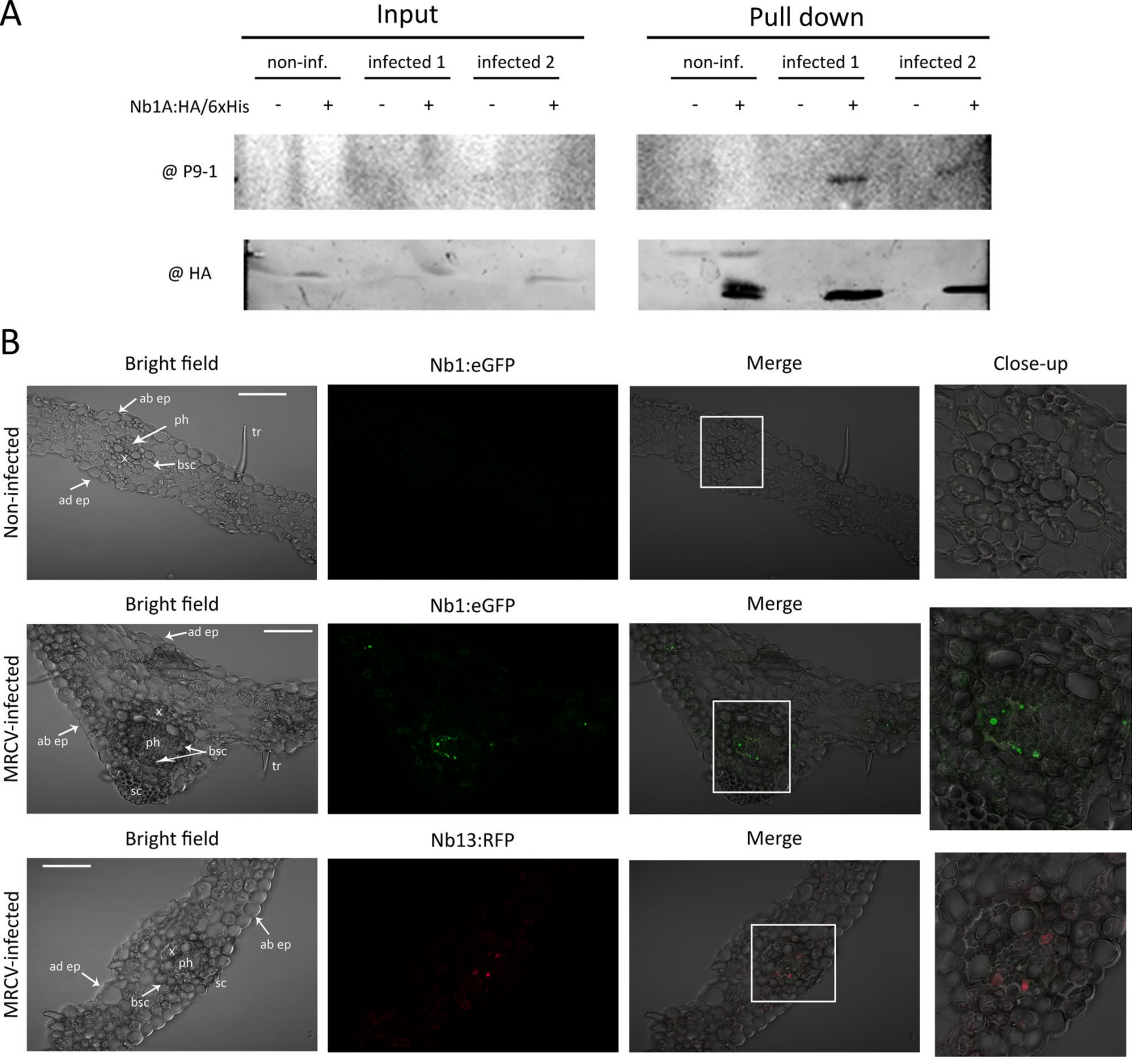
Immunodetection of MRCV P9-1 in infected plants using Nbs. (**A**) Assessment of the use of His-tagged Nb1 as a tool to pull down P9-1 present in infected maize plants. Total protein extracts from one non-infected (non-inf.) and two MRCV-infected (infected 1 and 2) maize plant leaves were incubated with 1 ug of purified Nb1:HA/6xHis and Ni–NTA resin. Extract samples before (input control) and after the pull-down were analysed. The presence of MRCV P9-1 (upper panel) was evidenced by western blot after a 10% SDS-PAGE using guinea pig anti-P9-1 sera as primary antibody and anti-guinea pig-HRP as secondary antibody. In turn, the presence of Nb1:HA/6xHis (lower panel) was detected by western blot after a 12% SDS-PAGE using mouse anti-HA as primary antibody and anti-mouse-AP as secondary antibody. Blots from (**A**) were cropped and grouped to simplify the Figure. (**B**) Confocal images after immunolocalisation of MRCV P9-1 in wheat leaf sections. Tissues were incubated with Nb1:eGFP or Nb13:RFP. Top panels correspond to non-infected plant samples whereas middle and bottom panels correspond to MRCV-infected samples at 35 days post-infection. In each case, images of the bright fields, green or red channels, the merging images and close-ups are shown. Phloem (ph), xylem (x), bundle sheath cells (bsc), sclerenchyma (sc), adaxial (ad) and abaxial (ab) epidermis (ep), and trichomes (tr) are marked. Scale bars = 100 µm.

To explore the utility of P9-1 specific Nbs as a fundamental research tool to unravel details of MRCV infection, we investigated Nb1 as a model to perform pull-down assays. Total proteins were extracted from MRCV-infected and non-infected maize leaves, and the extracts were incubated with purified Nb1. Taking advantage of the presence of HA and 6xHis tags fused to the Nb C-terminal end, the incubation of the plant extracts with Nb1 was performed in the presence of Ni–NTA resin to pull down the Nb and possibly its captured antigen. Then, the resin was washed, loaded with cracking buffer and analysed by western blot. The presence of P9-1 bound to the resin was monitored using guinea-pig anti-P9-1 antibodies (Supplementary Fig. S5) to avoid cross reaction with Nanobodies, while Nb could be detected with anti-HA antibodies. Results indicate that Nb1 captured *wild-type* P9-1, while no protein was detected in non-infected nor in MRCV-infected plant extracts incubated with the Ni–NTA resin alone (Fig. 5A). In conclusion, Nb1 is a useful tool for pull-down experiments, even to detect proteins present at low concentrations since MRCV and other fijiviruses only replicate in phloem cells and are therefore diluted when using total leaf extracts.

Immunofluorescence has been instrumental to study fijivirus dissemination within insect vectors as well as the structure and dynamics of viroplasm formation^40^. To explore the use of Nbs for immunodetection in infected leaves, we fused Nb13 to RFP and Nb1 to eGFP. For this assay we decided to use viruliferous planthoppers to artificially infect wheat since virus transmission is more efficient and this plant species is easier to handle^18^. Thirty-five days after artificial infection, thin leaves sections were cut and prepared for immunodetection. Non-infected wheat leaves were used as control. As expected from the well-known fijivirus phloem-limited replication^9^, fluorescence was exclusively detected at the sieve elements and companion cells of MRCV-infected leaves (Figs. 5B). Furthermore, no fluorescence was observed either in non-infected leaves nor in infected leaves without Nb1:eGFP or Nb13:RFP. Therefore, Nb:eGFP and Nb:RFP fusions were demonstrated to be versatile tools to detect MRCV P9-1 in plant tissues.

## Discussion

Global environmental changes are a major concern for plant pathologists since they influence the distribution of insect vector populations and affect both plant mechanisms of defence and pathogen’s virulence mechanisms^41^. In particular, a sixfold increase in the insect population of MRCV’s most important vector *Delphacodes kuscheli*^2^ was detected in spring 2018 compared to the last eight agricultural seasons^42^. At present, attempts to control Mal de Río Cuarto disease consist in crop-management practices that seek to prevent the peaks of insect vector populations from coinciding with the highly susceptible newly emerged seedlings^43^. Corn hybrids with different degrees of tolerance^44^ are also employed. However, these approaches fail to prevent virus circulation. Moreover, effective tools for Mal de Río Cuarto diagnosis and research have not yet been reported.

In plants, accurate virus detection is crucial to identify alternative host species of the virus and to precisely assess the prevalence of the disease since asymptomatic plants are frequently detected^34,35^. In plants, fijiviruses exclusively replicate in phloem cells, whereas in insects they accumulate first in the distal intestine and, after a latency period of about 2 weeks post-acquisition, they can also be detected in salivary glands^9^. Therefore, total virus concentration is relatively low both in whole plants and in insect vectors. Several methods have been used for diagnosis of other fijiviruses. Different types of ELISA tests were developed, most of them using polyclonal antibodies against the external capsid protein, peptides derived from it or against purified virus particles^45,46^. Later, monoclonal antibodies were raised against total plant tumours containing the virus and used for the development of sensitive antigen-coated-plate (ACP-ELISA) and dot-ELISA tests^47^. For MRCV diagnosis, a double antibody sandwich (DAS) ELISA test was developed using polyclonal antibodies raised against partially purified virus particles^3^. For detection, those antibodies are chemically conjugated to alkaline phosphatase, a process that is inefficient and that can lead to paratope obstruction, reduced detection yields and batch-to-batch variability^48^. In addition, methods relying on the detection of the virus genome such as RT-qPCR, LAMP or dsRNA genome hybridization have been developed as well for other fijiviruses^49,50^ and MRCV^51,52^. However, in general, ELISA tests are preferred over RT-PCR or LAMP analysis since they are user-friendly, fast, inexpensive (both in reagents and equipment) and less contamination-prone, allowing for high throughput screening of a large number of samples.

Due to their small size, their high affinity, specificity, stability, solubility, the ease with which they can be expressed in heterologous organisms, and their low cost of production, Nanobodies are increasingly being used in a plethora of applications^53,54^. In particular, they have been successfully employed for the detection of several animal diseases^31,32,36^, a plant virus^55^ and plant proteins^56^. Our work focused on the development of Nanobodies directed to the non-structural viral protein P9-1, which is the major viroplasm constituent^15–17^ of MRCV. Viroplasms of the *Reoviridae* family members are highly dynamic structures that are detected as early as 36 h post-infection in localized areas of the cytoplasm as small punctuate bodies that merge to larger bodies later in the infection^14,57^. Importantly, viroplasms are sites of viral mRNA synthesis, genome replication and nascent particles assembly^9^, and their composition and maturation has been extensively studied in animal reoviruses^58,59^ but to a much lesser extent in plant fijiviruses. To our knowledge, the Nanobodies developed in this work are the first to be obtained against a viroplasm component within the *Reoviridae* family. In this study, we were able to develop a sandwich ELISA using Nanobodies both as capture and detecting reagents. Even though MRCV is more abundant in maize roots^60^, for practical reasons we developed a diagnostic test able to detect the virus in leaves. The resulting ELISA showed high sensitivity and specificity to detect the virus presence and a low limit of detection of the target protein in maize leaves. Importantly, the ELISA was highly specific as no cross-reactivity was detected when testing several plant viruses commonly found in maize fields in Argentina or recombinant P9-1 from a closely related fijivirus.

Within the *Reoviridae* family, the quaternary structure of major viroplasm proteins is key to their function^39,61,62^. Having Nanobodies that could distinguish different quaternary structures of the viroplasm protein components could be an important asset to contribute with the study of viroplasm dynamics within infected cells. Along this line, for rotavirus, the development of two monoclonal antibodies (mAbs) that recognize distinct pools (a cytoplasmically dispersed and a viroplasmic) of NSP2 in infected cells was crucial to deepen into the modulation of viroplasm assembly^63,64^. With this in mind, and given the C-arm importance in VLS formation and P9-1 self-interactions^17,39^, we assessed if our selected Nbs were able to differentially recognize the deletion mutant P9-1 ΔC-arm and complete P9-1. As expected, the three Nbs bound P9-1 both in direct ELISA and western blot experiments. However, Nb1, Nb13 and Nb25 AP fusions bound differentially to P9-1 ∆C-arm, suggesting that Nb1 binds to an epitope that is not affected by the C-arm deletion, while Nb13 may target an epitope present either in the C-arm or in the final conformation of P9-1 multimers that are altered in the mutant, and Nb25 only bound to the reduced, denatured form of P9-1 ∆C-arm. Further studies are needed to precisely define the P9-1 binding sites in each case. Overall, we foresee the Nanobodies described in this work as promising tools to study P9-1 structural conformations.

Moreover, Nanobodies’ known superior sensitivity to mAbs due to their small size and convex shape allows them to access pockets and clefts of antigens that are inaccessible by regular antibodies. This property and their superb versatility paved their use for the development of ultra-high affinity reagents for purification^28,65^. In this regard, our work provides an exciting biotechnological tool that is efficient to immunocapture P9-1 present in naturally infected plants and that could be used, for example, for the identification of host proteins associated to MRCV viroplasms by pull-down followed by mass spectrometry analysis.

The major drawbacks for the use of polyclonal antibodies conjugated with fluorochromes such as FITC or rhodamine to immunodetect viral proteins in plant or insect infections are their high costs, the time-consuming use of secondary antibodies and the sometimes difficult accession to target proteins. The development of Nbs against P9-1 fused to fluorescent proteins allowed us to specifically detect the virus presence in the phloem cells of plant leaves and could also be used to follow MRCV replication within the different sections of the delphacid intestine as reported for other fijivirus^66^.

Finally, Nanobodies targeting proteins from diverse viruses such as HIV^67^, Influenza A^68^, Norovirus^69^, coronaviruses^70^, were shown to have antiviral activity. In particular, Nbs targeting rotavirus (a reovirus as well as MRCV) VP6 middle layer capsid protein display protective effects against rotavirus induced diarrhea^71^. Furthermore, this strategy has been successfully used to control the plant viruses Broad-bean mottle virus (BBMV, Bromovirus)^72^ and Grapevine fan leaf virus (GFLV, Nepovirus)^73,74^.

Overall, the Nbs obtained in this work allowed the development of a sensitive and specific sandwich ELISA to detect MRCV, constitute innovative biotechnological tools for fundamental research as shown in pull-down and immunolabelling assays, and could as well contribute to the design of novel biotechnological antiviral strategies.

## Methods

All methods were carried out in accordance with relevant international guidelines and regulations.

### Antigen preparation

A pRSET P9-1 construct containing the full MRCV P9-1 coding sequence (GenBank: FJ890851.1)^15^ was employed for MRCV P9-1 expression. Protein was purified by IMAC as described^15^ followed by size-exclusion chromatography (SEC) using a Superdex 200 16/60 column (GE Healthcare, USA) and running buffer (10 mM Tris–HCl pH 7.6, 25 mM NaCl) at a flow rate of 1 ml/min.

P9-1 ΔC-arm^17^ was expressed and purified using the same protocols as for MRCV P9-1.

### Llama immunisation

A young female llama was immunised with four doses of 100 µg P9-1 recombinant protein each, mixed with complete Freund’s adjuvant on day 0, and with incomplete Freund’s adjuvant on days 15, 29 and 42. Peripheral blood samples were collected to monitor the llama antibody response by ELISA.

At day 57, 200 ml of whole blood were collected to isolate peripheral blood mononuclear cells (PBMCs) by a Ficoll density gradient using Hystopaque^®^-1077 solution. Cells were maintained at − 20 °C in RNA later until use. Llama handling, inoculation, and sample collection were conducted under supervision of veterinarians. The study and animal welfare protocols were approved by INTA Institutional Animal Care and use Committee IACUC^75^. The study was in compliance with the ARRIVE guidelines^76^.

### Library construction and enrichment of P9-1-specific Nanobodies from the VHH library by phage display

Library construction was performed as described^77^. PCR products containing Nb coding sequences were purified and directionally cloned through digestion with PstI and NotI restriction enzymes into pMECS phagemid vector, and electroporated into competent *E. coli* TG1 cells. Specific Nbs from the library were enriched after three rounds of phage display selection against 10 µg of immobilized recombinant P9-1, as described^77^.

### Golden-gate plasmid construction

Cloning of Nbs fused to alkaline phosphatase (Nb:AP), enhanced green fluorescent protein (Nb:eGFP) and Tag-red fluorescent protein (Nb:RFP), was carried out using Golden Gate^78^. Donor vectors coding for C-terminal 6xHis-tagged AP, eGFP or RFP flanked by SapI restriction sites, destination vectors allowing expression in bacterial periplasm (pETGGp) or cytoplasm (pETGGc) as well as primers design, were kindly provided by Dr. V. Poignavent and Dr. C. Ritzenthaler (IBMP-CNRS). Nbs coding sequences were PCR amplified using primers 767 F and 768 R that have flanking SapI restriction sites. For Nb:AP cloning, expression vector pETGGp, donor vector and insert were mixed in the reaction at a molar ratio of 1:3:6, respectively, with 2 µl of Cut Smart 10× Buffer, 2.5 mM ATP, 2 U of T4 DNA ligase, 5 U of SapI and water up to 20 µl. For Nb:eGFP or Nb:RFP cloning, the pETGGp and AP donor vector were respectively replaced by pETGGc and eGFP or RFP donor vector. For bivalent Nbx2:eGFP cloning, Nb coding sequence was PCR amplified using two sets of primers: 767 F with Nb(G4S)3 and (G4S)3Nb with 768 R, these primers adding SapI flanking sequences and coding for a flexible linker between each Nb. PCR products were added in the reaction mix, with an expression vector to donor vector to inserts molar ratio of 1:3:6:6. Golden Gate reactions were performed by ten cycles of 10 min at 37 °C for digestion, 10 min at 18 °C for ligation, 1 h at 18 °C for ligation of the undigested products, 10 min at 50 °C and 10 min at 80 °C for SapI and T4 DNA ligase inactivation. As a result, pETGGp Nb:AP, pETGGc Nb:eGFP, pETGGc Nb:RFP and pETGGc Nbx2:eGFP vectors were obtained.

### Nanobodies expression and purification

The pMECS clones, able to express the selected Nbs fused to 6xHis and HA tags (Nb:HA/6xHis) for purification and detection, were transformed into *E. coli* WK6 cells as described^77^. For Nb:AP expression, *E. coli* BL21 strain carrying pETGGp Nb:AP was grown in TB supplemented with 0.08% glucose at 37 °C until OD_600nm_ reached 0.8–1.2 and induced with 1 mM IPTG for ON expression at 20 °C. For Nb:HA/6xHis and Nb:AP protein extraction, cells were harvested by centrifugation and periplasmic proteins were extracted by osmotic shock with TES (0.2 M Tris–HCl pH 8.0, 0.5 M sucrose, and 0.5 mM EDTA) and a ¼ TES dilution. Nb:eGFP, Nb:RFP and Nbx2:eGFP expression was carried out as for Nb:AP but *E. coli* SHuffle strain was employed and grown at 30 °C until induction. After culture centrifugation, bacterial cells were suspended in a buffer containing 100 mM Tris HCl pH 7.5, 300 mM NaCl and 5% glycerol and DNA was sheared by sonication. Next, cultures were centrifuged and supernatants collected. All Nbs protein extracts were subjected to IMAC purification as described^77^. Nb:AP, Nb:eGFP, Nb:RFP and Nbx2:eGFP buffer was exchanged by storage buffer (25 mM Tris HCl pH 8.0, 100 mM NaCl). The Nbs fusions concentrations were measured with a spectrophotometer (NanoDropTM 1000, Thermo Fisher Scientific, USA). After IMAC, Nb:HA/6xHis were concentrated up to 0.5 ml and further purified by SEC using a Superdex 75 10/300 GL column on an ÄKTA Explorer chromatography system (GE Life Sciences, USA). These purified Nbs were then employed for SPR affinity measurements.

### Surface plasmon resonance (SPR)

The kinetic affinity parameters of the Nbs for P9-1 were determined by surface plasmon resonance (BIACORE-T200, GE Healthcare). P9-1 was coupled to a CM5 sensor chip following manufacturer’s recommendations. Serial dilutions of anti-P9-1 Nbs in HBS (10 mM HEPES pH 7.4, 150 mM NaCl, 0.005% Tween-20, 3.4 mM EDTA) were flown at 20 μl/min and 25 °C over the sensor layer. The association step was 150 s, the dissociation step was 600 s. The rate kinetic constants were calculated by a mathematical fitting of a 1:1 binding model using the BIACORE Evaluation software (GE Healthcare, USA), and the k_d_/k_a_ ratio was used to determine the equilibrium dissociation constant (K_D_).

### Development, analytical and diagnosis validation of Nanobody-based ELISAs to detect MRCV in plants

Plates were coated in triplicates with 100 µl of 5 ng/µl of purified Nb:eGFP fusions and incubated ON at 4 °C. Next, each well was blocked with of 5% skimmed milk for 1 h at 37 °C. For protein extraction, 100 mg of grinded plant tissues were dissolved in 500 µl of extraction buffer (PBS containing 2% Polyvinylpyrrolidone (PVP40), 0.05% Tween 20 and 2% skimmed milk), vortexed, incubated on ice for 20 min and centrifuged. Hundred µl of plant protein extracts were added and incubated for 1 h at 37 °C. Then, 100 µl of 0.5 ng/µl of Nb:AP fusions in PBS were added to each well and incubated at RT for 1 h. After each step, plates were washed three times with PBS containing 0.05% Tween 20. Finally, plates were developed by adding 100 µl of 1 mg/ml of pNPP and absorbance was read at 405 nm after 1 h.

The capture antibody’s ability to bind P9-1 was compared between 100 µl of equimolar amounts of Nb13:eGFP and Nb13 × 2:eGFP. Next, 1 mg/ml and 2 mg/ml pNPP concentrations were compared. To further optimize the sandwich ELISA protocol, plates were coated with 100 µl of 5 ng/µl of Nb13 × 2:eGFP for 4 h at 37 °C, plant extracts were incubated ON at 4 °C and 1.25 ng/µl Nb1:AP was dissolved in extraction buffer. Subsequently, a checkerboard titration was performed, where Nb13 × 2:eGFP and Nb1:AP were evaluated in  fivefold dilutions.

The limit of detection was determined using non-infected plant extracts spiked with purified recombinant P9-1 in twofold dilutions. Every sample was measured in triplicates. Data was fitted to a 4-parameter logistic equation:

$$f \left[x, \left(b, d, c, e\right)\right]=\frac{d-c}{1 + {e}^{\left[b \left(\mathrm{log}\left(x\right)-\mathrm{log}\left(e\right)\right)\right]}}$$

where $d$ is the response to infinite analyte concentration, $c$ is the response at zero analyte concentration, $b$ is the slope factor, $x$ is the analyte concentration and $e$ is the half maximal effective concentration (EC_50_).

Analytical specificity was determined on maize infected either with MYSV, MDMV or SCMV and on wheat infected with WSMV. Protein extraction and ELISA procedures were executed as explained before. Recombinant protein ELISA was performed by adding 500 ng of either MRDV P9-1 or MRCV P9-1 to non-infected maize extracts and the sandwich ELISA procedure was carried out as detailed before.

For assay diagnosis validation (determination of cut-off and their associated diagnosis sensibility and specificity), a total of 212 plants were analysed for virus presence by RT-PCR (see Supplementary Methods) and these samples were further processed using the final ELISA protocol.

### Statistical analysis

Maximized Youden's index was chosen for optimal cut-off selection^79^. Statistical analyses were performed using GraphPad Prism 7 software (GraphPad Software, Inc. LA Jolla, CA, USA). Differences in values between study groups were assessed by analysis of variance (ANOVA) and Tukey’s multiple comparison test and p-values < 0.05 were considered statistically significant. ROC curve and cut-off analysis, were done using R 3.6.3^80^ and the OptimalCutpoints package^79^.

### Western blot of denaturing and native-PAGE

The P9-1 and P9-1 ΔC-arm proteins separated by denaturing and native-PAGE were analysed by western blot using Nb:AP. For denaturing, reducing conditions, 10 µg of purified P9-1 and P9-1 ΔC-arm were boiled for 5 min in loading buffer (62.5 mM Tris–HCl pH 6.8, 2.5% SDS, 0.002% Bromophenol Blue, 5% β-mercaptoethanol and 10% glycerol), and subjected to 10% SDS-PAGE. For native, non-reducing conditions, 5 µg of purified P9-1 and P9-1 ΔC-arm were loaded with loading buffer without β-mercaptoethanol and SDS, and subjected to 8% native-PAGE. Then, native and denatured proteins were transferred to a nitrocellulose membrane, proper protein transfer was assessed by Ponceau S staining, and analysed by western blot, using 10 ng/µl of Nb:AP in Tris Buffer Salt (150 mM NaCl, 20 mM Tris, pH 7.6) with 5% skimmed milk. Detection was performed using NBT-BCIP reagents.

### Pull down of P9-1 from infected plant tissues

Hundred µl containing total protein extracts from non-infected or MRCV-infected maize plants were incubated with 1 µg of purified Nb1:HA/6xHis. Fifty µl of Ni–NTA resin was added to the incubation, and after washing, the presence of bound P9-1 was assessed by western blot. P9-1 was detected using the guinea pig anti-P9-1 sera (see Supplementary Methods) at a 1/1600 dilution as primary antibody and 1/3000 goat anti-guinea pig-HRP as secondary antibody. Nbs were detected with 1/500 mouse anti-HA as primary antibody and 1/5000 anti-mouse-AP as secondary antibody.

### Immunolocalisation of P9-1 in infected plant tissues using Nanobodies fused to eGFP or RFP

Cross sections of wheat leaves either from non-infected or MRCV-infected plants, were processed for immunofluorescence confocal laser scanning microscopy (iCLSM) as described^81^, with modifications. Leaf sections were fixed in 4% paraformaldehyde in 0.1 M phosphate buffer pH 7.4 and 0.1% Triton X 100 ON at 4 °C, washed in PBS with 0.1% Triton X-100 and immediately hand-sectioned fine enough to achieve transparency in PBS under a stereo microscope (Nikon SMZ 800 N). Semi-thin sections were mounted on microscope slides and immersed in blocking buffer (PBS, 0.1% Triton X-100, 10% normal goat serum) for 30 min in wet chamber at RT and incubated for 2 h at RT in wet chamber with Nb1:eGFP or Nb13:RFP diluted in incubation buffer (PBS, 0.1% Triton X-100, 1% normal goat serum) at a final concentration of 1 ng/µl for Nb1:eGFP and 2 ng/µl for Nb13:RFP. Finally, specimens were extensively washed in PBS and 0.1% Triton X-100 and covered in 50% glycerol prior to confocal imaging (see Supplementary Methods).

All oligonucleotide sequences are listed at Supplementary Table S1 (see Supplementary Methods).

### Statement

Plant sample collection and handling was performed by Dr. Mariana del Vas on January 2018 at Río Cuarto County, Córdoba Province, Argentina, following institutional, national, and international guidelines and legislation. Permission for collecting the detached leaves was obtained from the owner of the field.

## Supplementary Information

Supplementary Information.
