# Supplementary material for: Development of Nanobodies against Mal de Río Cuarto virus major viroplasm protein P9-1 for diagnostic sandwich ELISA and immunodetection
Source: Sci Rep. 2021 Oct 8;11:20013. doi: 10.1038/s41598-021-99275-z (PMC8501053; doi:10.1038/s41598-021-99275-z)
Supplement: Supplementary file 1 — Supplementary Information. [file 41598_2021_99275_MOESM1_ESM.pdf]

Development of Nanobodies against Mal de Río Cuarto virus major viroplasm protein P9-1 for diagnostic sandwich ELISA and immunodetection

Gabriela Llauger[+,a], Demián Monti[+,a], Matías Adúriz[b], Ema Romão[c], Analía Delina Dumón[d,e], María Fernanda Mattio[d,e], Andrés Wigdorovitz[b], Serge Muyldermans[c,f], Cécile Vincke[c,g], Viviana Parreño[b], Mariana del Vas[a,*]

Supplementary Methods

RNA extraction, cDNA synthesis and PCR analysis

For diagnosis, total RNA from maize leaves was extracted using TranZol (TransGen Biotech, China) followed by cDNA synthesis using random primers and MMLV (Thermo Fisher Scientific, USA), according to manufacturer's instructions. Finally, diagnostic PCR was performed using virus-specific primers annealing either at S1 (S1 up and S1 low) or S9 (18b up and 45a low) genome segments. Ten MRCV-infected and ten MRCV-non-infected samples were separately pooled and used for sandwich ELISA optimization.

MRDV P9-1 cloning, expression and purification

Total MRDV dsRNA purified from infected maize was kindly provided by Dr. T. Candresse[1]. For MRDV P9-1 cloning, total cDNA was synthetized using Super Script III™ and random primers (Thermo Fisher Scientific, USA), following manufacturer's instructions. Next, MRDV P9-1 coding sequence was PCR amplified using specific primers MRDV up P9-1 s/ATG and MRDV low P9-1 c/Stop, cloned into pGEMT-Easy vector (Promega, USA), sequenced, and used as template for a new PCR with primers SapI P9-1 MRDV up 6xHis and SapI P9-1 MRDV low which added flanking SapI restriction sites (underlined) and an N-terminal 6xHis tag to the MRDV P9-1 coding sequence. Finally, a Golden Gate assembly reaction was performed as detailed previously, using the purified PCR products and pETGGc as destination

vector in a 3:1 molar ratio. Expressions and purifications were performed using the same protocols as for MRCV P9-1.

Virus and insect handling

MRCV (isolate MRCV-2008) and the proposed cytorhabdovirus Maize yellow striate virus MYSV (isolate MYSV-2013) were previously described[2–4]. These isolates were maintained on wheat (cv. ProINTA Federal for MRCV and cv. Biointa 3005 for MYSV) by serial vector transmissions using *Delphacodes kuscheli*[3,5] from a colony at the Vector's Laboratory of Instituto de Patología Vegetal-Centro de Investigaciones Agropecuarias-Instituto Nacional de Tecnología Agropecuaria (IPAVE-CIAP-INTA), as described[5]. Second instar *D. kuscheli* nymphs fed on MRCV-infected wheat plants were used for 1:1 transmission assays[5]. Nymphs fed on non-infected wheat were used as control treatments.

Wheat streak mosaic virus (WSMV, *Tritimovirus*, *Potyviridae*) isolate was obtained from infected wheat plants collected in 2014 in Marcos Juárez (Province of Córdoba, Argentina) and maintained in wheat (cv. Biointa 3005) at the Vector's Laboratory (IPAVE-CIAP-INTA), by consecutive transmissions using *Aceria tosichella* as vector[6].

Maize dwarf mosaic virus (MDMV, *Potyvirus*, *Potyviridae*) and Sugarcane mosaic virus (SCMV, *Potyvirus*, *Potyviridae*) maize-infected samples were kindly provided by Dr. Fabián Giolitti (IPAVE, INTA). Both isolates were initially collected at the Province of Córdoba, Argentina.

Plant material

Cultivated maize (*Zea mays*) samples were collected in Río Cuarto, Province of Córdoba, Argentina, where MRCV is endemic. Three fully developed leaves of symptomatic and asymptomatic maize plants were cut and kept in dry ice. Next, each sample was grinded with liquid nitrogen using a mortar and stored at -80 °C until use.

Direct ELISA for detection of recombinant MRCV P9-1

Plates were coated at 4 °C ON with 100 µl of P9-1 or P9-1 ΔC-arm in two-fold dilutions in triplicates. After blocking residual protein binding sites with 5% skimmed milk, 100 µl of 1.25 ng/µl of Nb:AP dissolved in extraction buffer were added to each well and incubated at 37 °C for 1h. Finally, ELISAs were developed by adding 100 µl of 2 mg/ml of pNPP and absorbance was read at 405 nm.

Production of polyclonal antisera against MRCV P9-1

To raise polyclonal antibodies against P9-1, three guinea pigs received two subcutaneous injections with 53 µg of purified recombinant P9-1, one at day 0 with complete Freund's adjuvant, and the second at day 28 with incomplete Freund's adjuvant. The immune sera titres were monitored by direct ELISA at days 0, 16 and 34, while the antiserum was collected at day 40 after the first immunisation. The ELISAs were performed as indicated in the llama immunisation section. Sera from the three guinea pigs were pooled, used at two-fold serial dilutions from 1/800 to 1/51200, and detection was performed with 1/3000 dilution of goat anti-guinea pig-HRP (KPL, Sera Care, USA). For sera titration at day 40, four-fold serial dilutions from 1/3200 to 1/13107200 of each serum were employed.

Guinea pigs handling, inoculation, and sample collection were conducted by supervision of veterinarians under animal welfare protocols and guidelines approved by INTA Institutional Animal Care and use Committee IACUC[7] and in compliance with the ARRIVE guidelines[8]. All methods were carried out in accordance with relevant international guidelines and regulations.

Confocal imaging

Samples were analysed in a Nikon Eclipse CS1i confocal microscope using a Plan Apo VC 20.0x objective (AN 0.75). eGPF, in Nb1:eGFP, was excited with the 488-nm Argon laser line running at 10% and the emission was collected through DM 480 and BA 515/30 filters. For the observation of the

Nb13:RFP, RFP was excited with a Helium-Neon (He-Ne) laser (543 nm) and the emission was collected through DM 545 and BA 605/75 filters. The laser scan for the acquisition of the definitive images was performed with a residence time of 5 µs and a pinhole opening of 30 µm. Bleeding between channels was avoided by acquiring the images in the “Frame lambda” mode of the EZ-C1 acquisition software (Silver Version 3.91). In this mode, each channel is acquired sequentially, starting with the lowest energy laser (He-Ne, in this case). This laser was also used to acquire images of the transmitted light channel.

In addition, “sham” samples were processed without Nb1:eGFP nor Nb13:RFP in order to subtract autofluorescence background. Therefore, the gain of each channel was set to a minimum with these samples and this configuration was used for the acquisition of all the images, with a resolution of 1024x1024 dots per inch. No post-image processing was performed.

Supplementary Figures

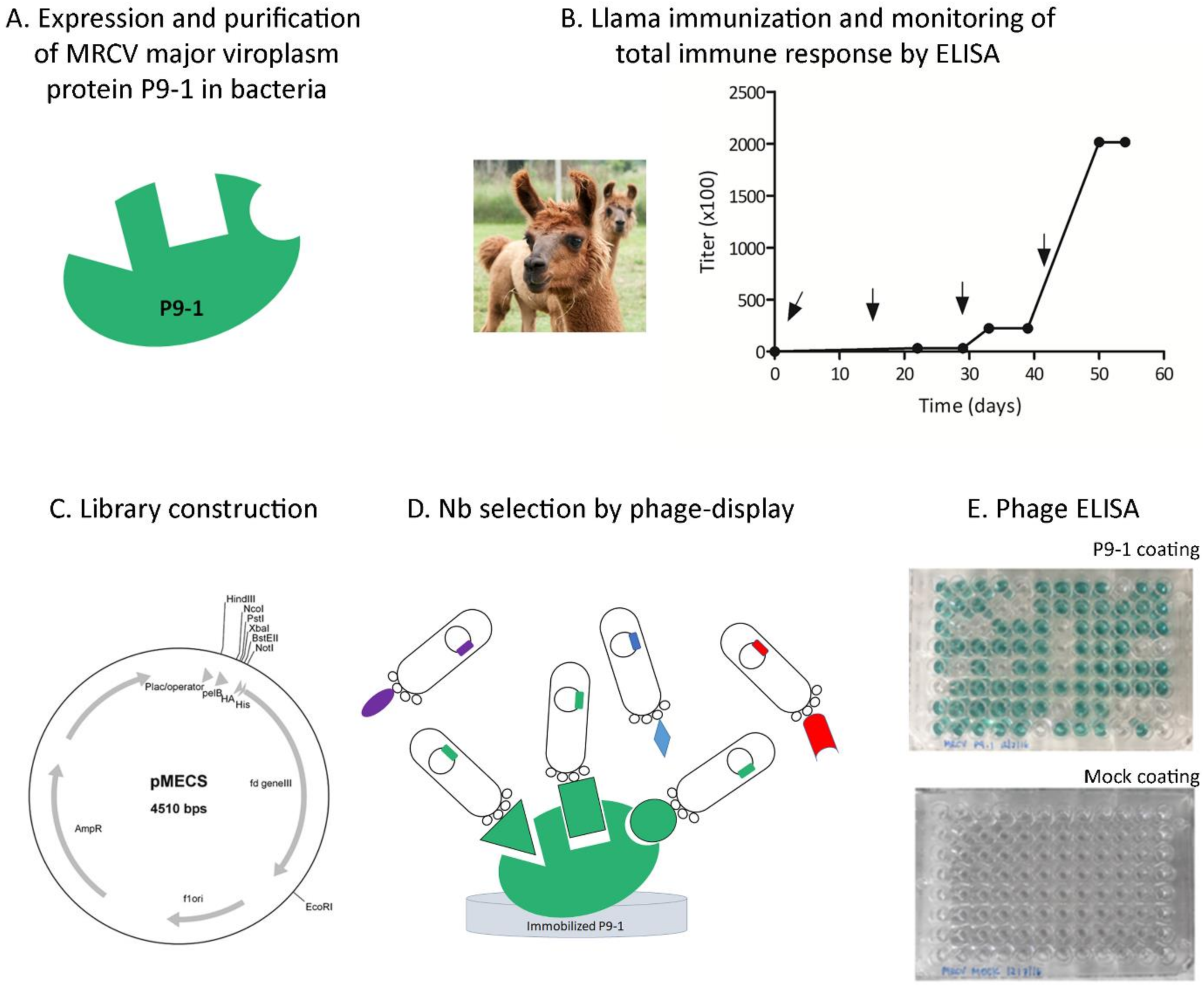

Supplementary Figure S1. Schematic representation of the different steps to obtain and select P9-1 specific Nanobodies by phage-display. (**B**) Llama picture source: INTA. Arrows in (**B**) indicate the time of immunisation with purified P9-1. (**C**) pMECS plasmid image source: Vincke, C. *et al*[9].

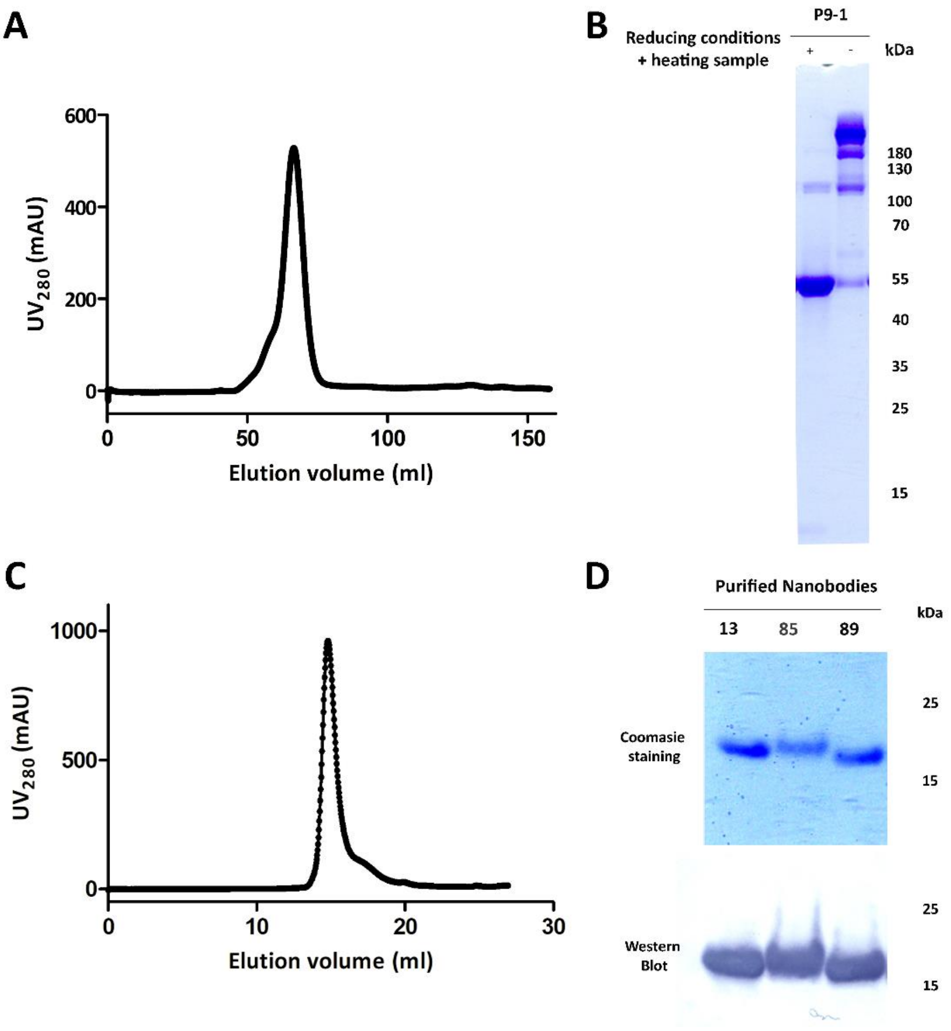

Supplementary Figure S2. Purification and assessment of MRCV P9-1 and Nanobodies purity. (**A**) After IMAC purification, P9-1 was subjected to size exclusion chromatography (SEC). (**B**) Purified P9-1 was loaded on a 12 % SDS-PAGE and stained with Coomassie blue. After boiling under reducing conditions, P9-1 migrates mostly as a monomer, while in non-reducing and non-heating conditions, the protein homomultimerizes. (**C**) Similarly, after IMAC purification SEC was performed to the eight selected Nanobodies with a S75 column (a chromatogram for Nb25 is shown as an example). (**D**) The Nanobodies' purity was also evaluated by a 12% SDS-PAGE followed by Coomassie blue staining (upper panel) and western blot detection with a mouse anti-His antibody and goat anti-mouse IgG-HRP (lower panel). As an example Nbs 13, 85, and 89 are shown. Blots from **B** and **D** were cropped and grouped to simplify the figure**.**

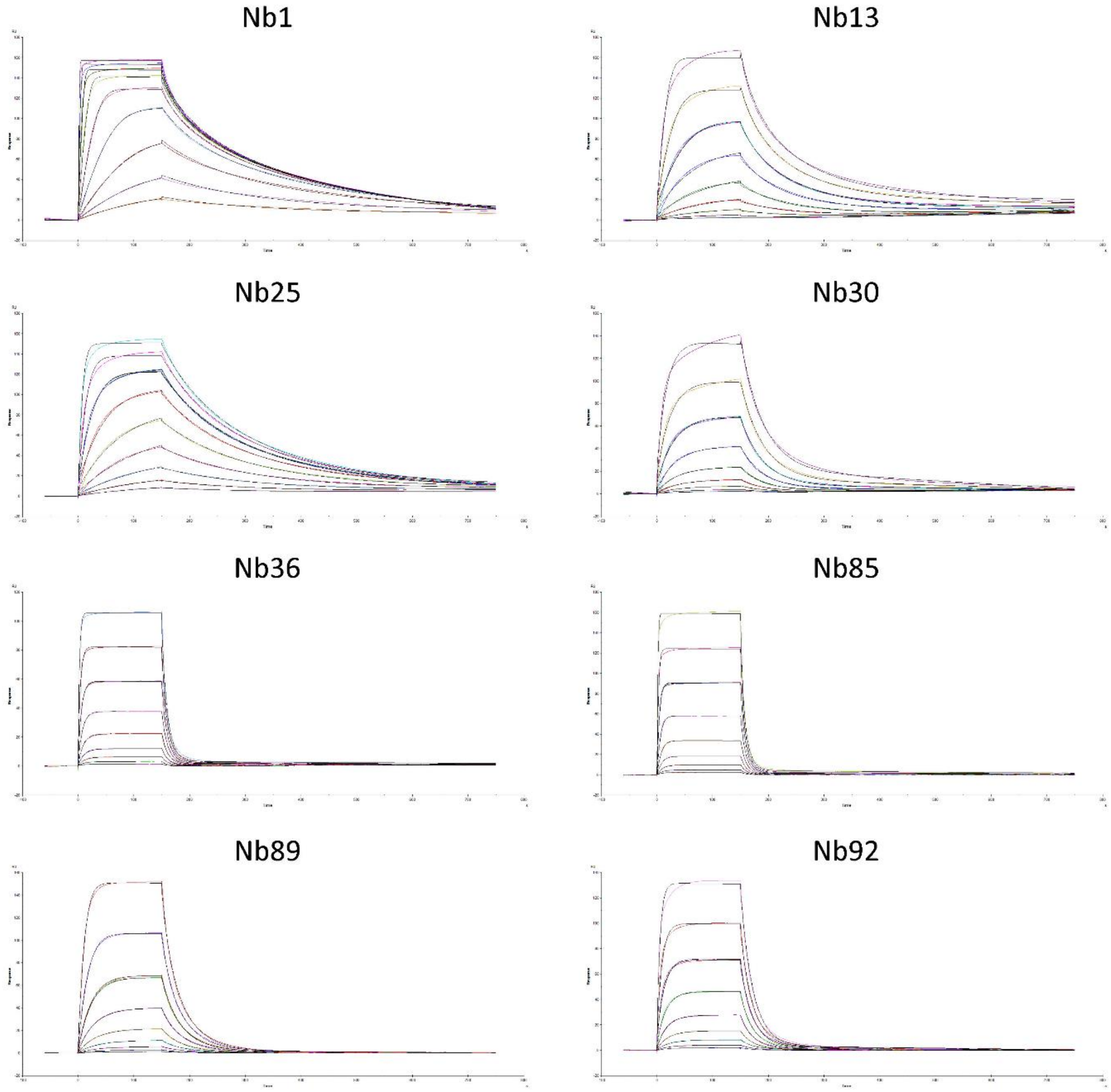

Supplementary Figure S3. SPR sensorgrams of the interaction between the eight selected Nbs and P9-1. Two-fold diluted series (from 250 nM to 1.953 nM) of the selected Nbs against P9-1 were employed for kinetics measurements. The black curves indicate the 1:1 binding model mathematical fit for each dilution.

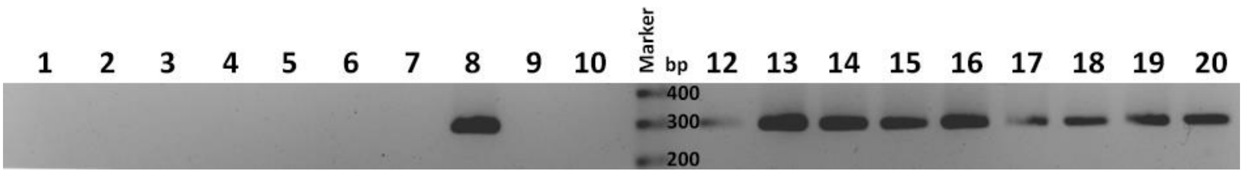

Supplementary Figure S4. RT-PCR detection of MRCV in maize leaf samples. Amplification products of 304 bp using primers annealing on MRCV genomic segment S1 are shown. Lanes 1 to 10 correspond to the analysis of asymptomatic maize plants, lane 11 is 100 bp DNA molecular weight marker and lanes 12 to 20 represent symptomatic samples. Nineteen of the 212 samples analysed are shown. Asymptomatic sample in lane 8 is infected with MRCV. The gel was cropped to simplify the Figure.

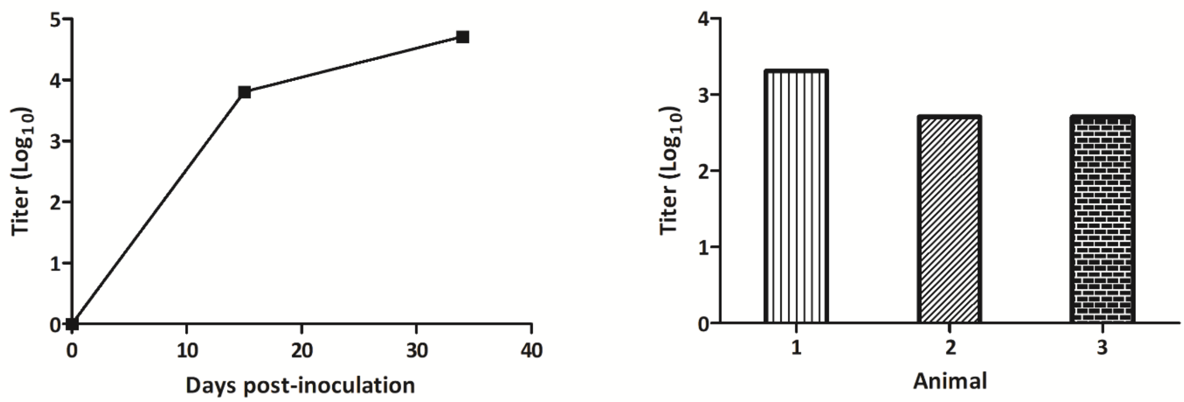

Supplementary Figure S5. Development of guinea pig polyclonal antibodies against MRCV P9-1. (**A**) Guinea pigs P9-1 antibody response through the course of immunisation. Blood was sampled at 0, 15 and 34 days post-inoculation (dpi) (n=3), pooled, and analysed by direct ELISA. (**B**) At 40 dpi, anti-P9-1 antibodies of each animal were titrated by direct ELISA.

Name	Sequence 5´-3´
767 F	ACTTGGCTCTTCTATGCAGGTGCAGCTGCAGGAGTCTGGRGGAGG
768 R	ATGATGCTCTTCTGGAGGAGACGGTGACCTGGGT
Nb(G4S)3	TAAGCTCTTCGGCCGCTGCCGCCGCCGCCGCTGCCGCCGCCGCCGCTGCCGCCGCCGCCTGAGGAGACGGTGAC
(G4S)3Nb	TAAGCTCTTCGGGCGGCGGCGGCGGCAGCGGCGGCGGCGGCAGCGGCGGCGGCGGCAGCCAGGTGCAGCTGCAG
MRDV up P9-1	GCAGACCAAGAGCGGAGAAC
MRDV low P9-1 c/Stop	TCAAACGTCAAGCTTCAAAG
SapI P9-1 MRDV up 6xHis	TAAGCTCTTCGATGCACCACCACCACCACCACGCAGACCAAGAGCGG
SapI P9-1 MRDV low	TAAGCTCTTCGTCAAACGTCAAGCTTCAA
S1 up	ATGGTGGAACAAAGTTTCAGTAGAT
S1 low	TAATCTCTTCGGCATTCGCA
18b up	GATAAAGATACAGGAGCGGTTCA
45a low	CTCGTTTGTTATCTGGTTTT

Supplementary Table S1. Sequences of the oligonucleotide primers employed throughout the work. Underlined sequence indicates SapI restriction site.

Supplementary Information: Full-length gels and blots

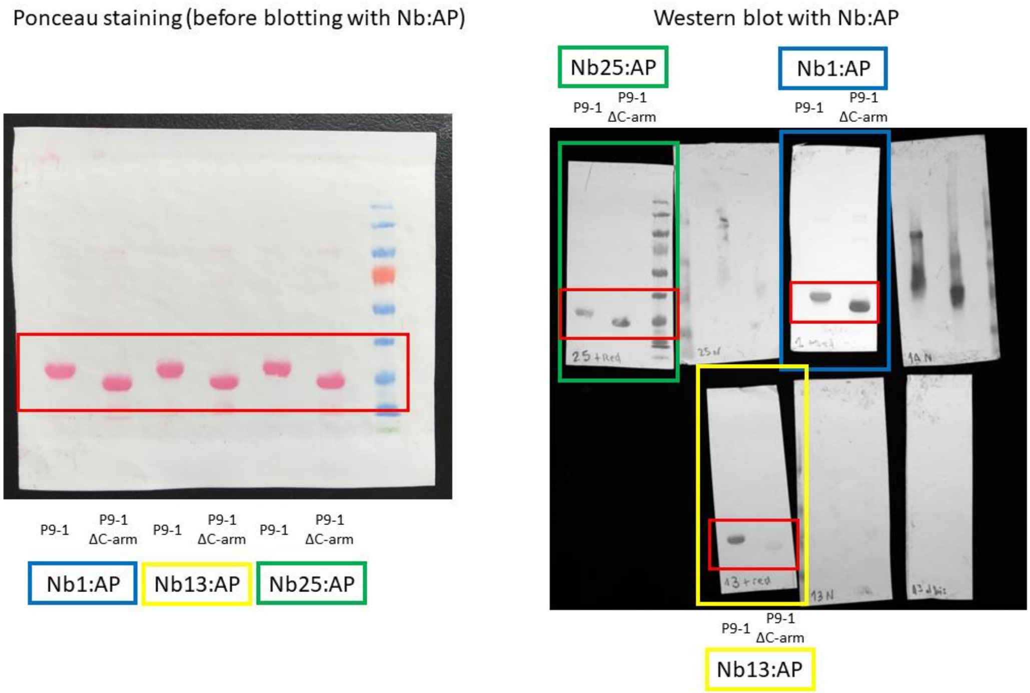

Figure 4C: The regions of the original blots used in main figures were denoted by red boxes and each of the Nb:AP fusions used were marked with a different color.

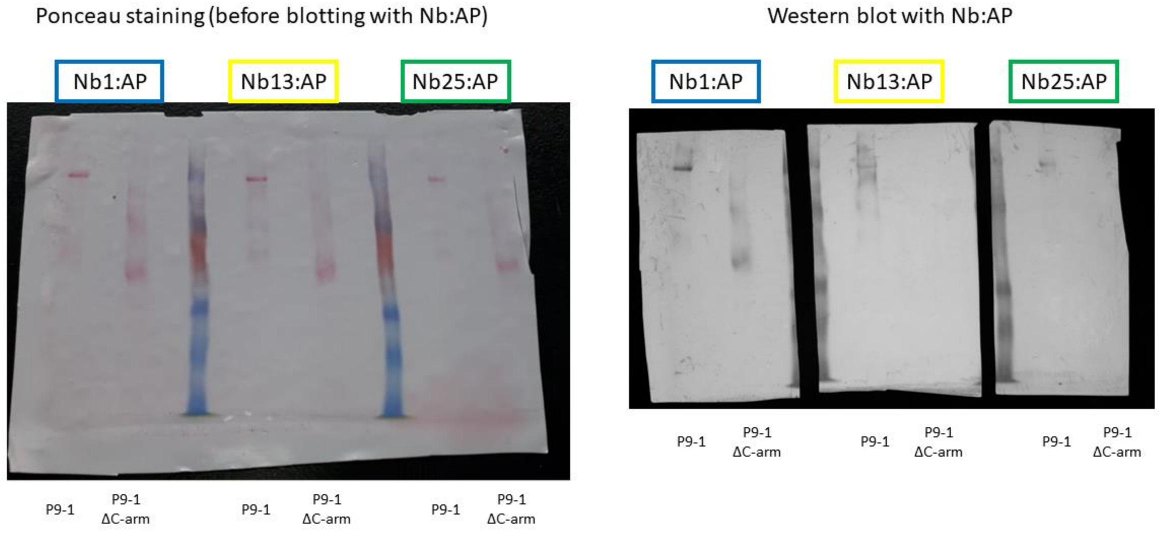
Ponceau staining (before blotting with Nb:AP)
Nb1:AP
Nb13:AP
Nb25:AP
P9-1
P9-1
ΔC-arm
P9-1
P9-1
ΔC-arm
P9-1
P9-1
ΔC-arm
Western blot with Nb:AP
Nb1:AP
Nb13:AP
Nb25:AP
P9-1
P9-1
ΔC-arm
P9-1
P9-1
ΔC-arm
P9-1
P9-1
ΔC-arm

Figure 4D

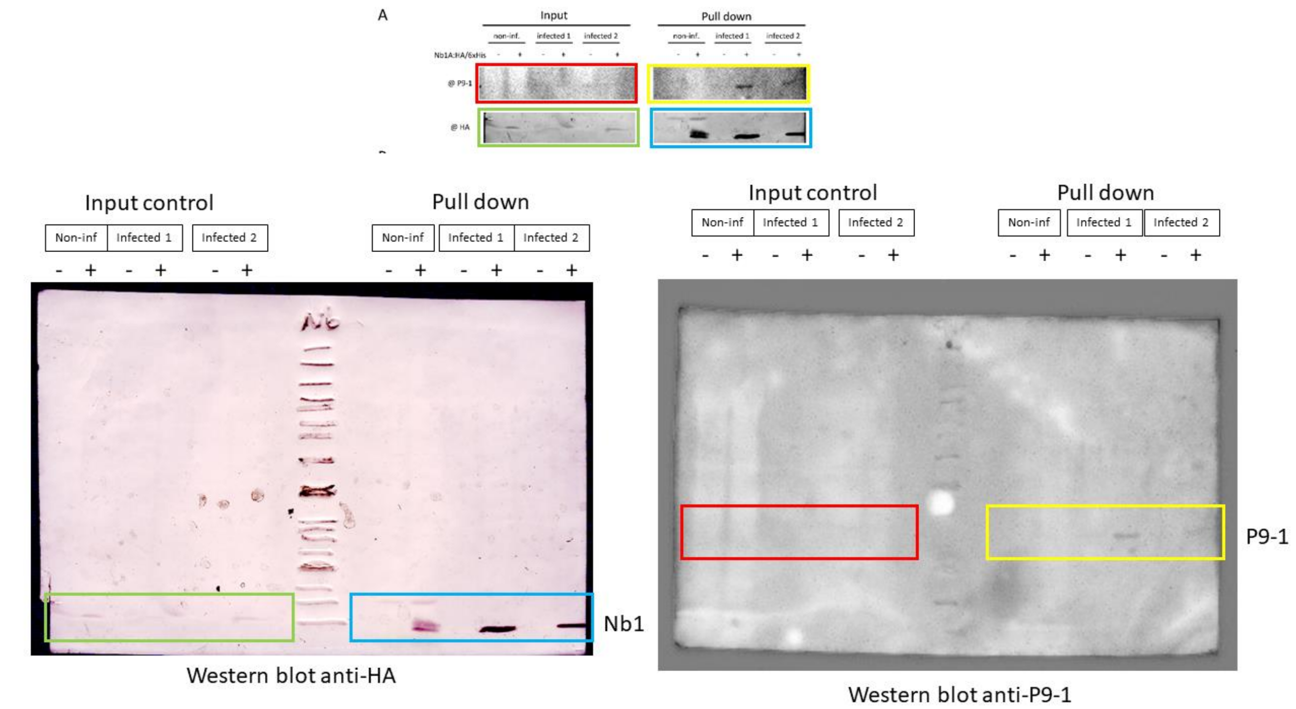

Figure 5A: The regions of the original blots used in main figures were denoted by colored boxes.

B. Purified P9-1

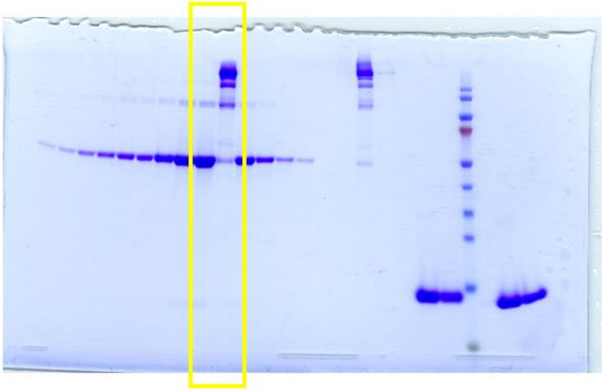

D. Purified Nanobodies

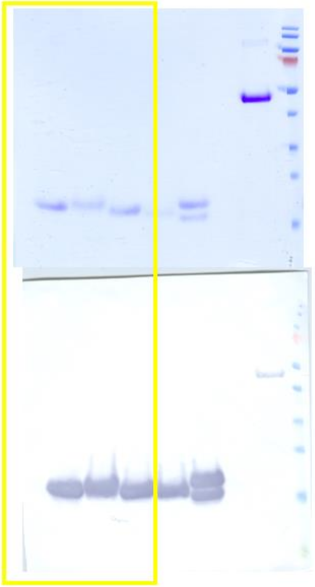

Supplementary Figures S2, panels B and D.

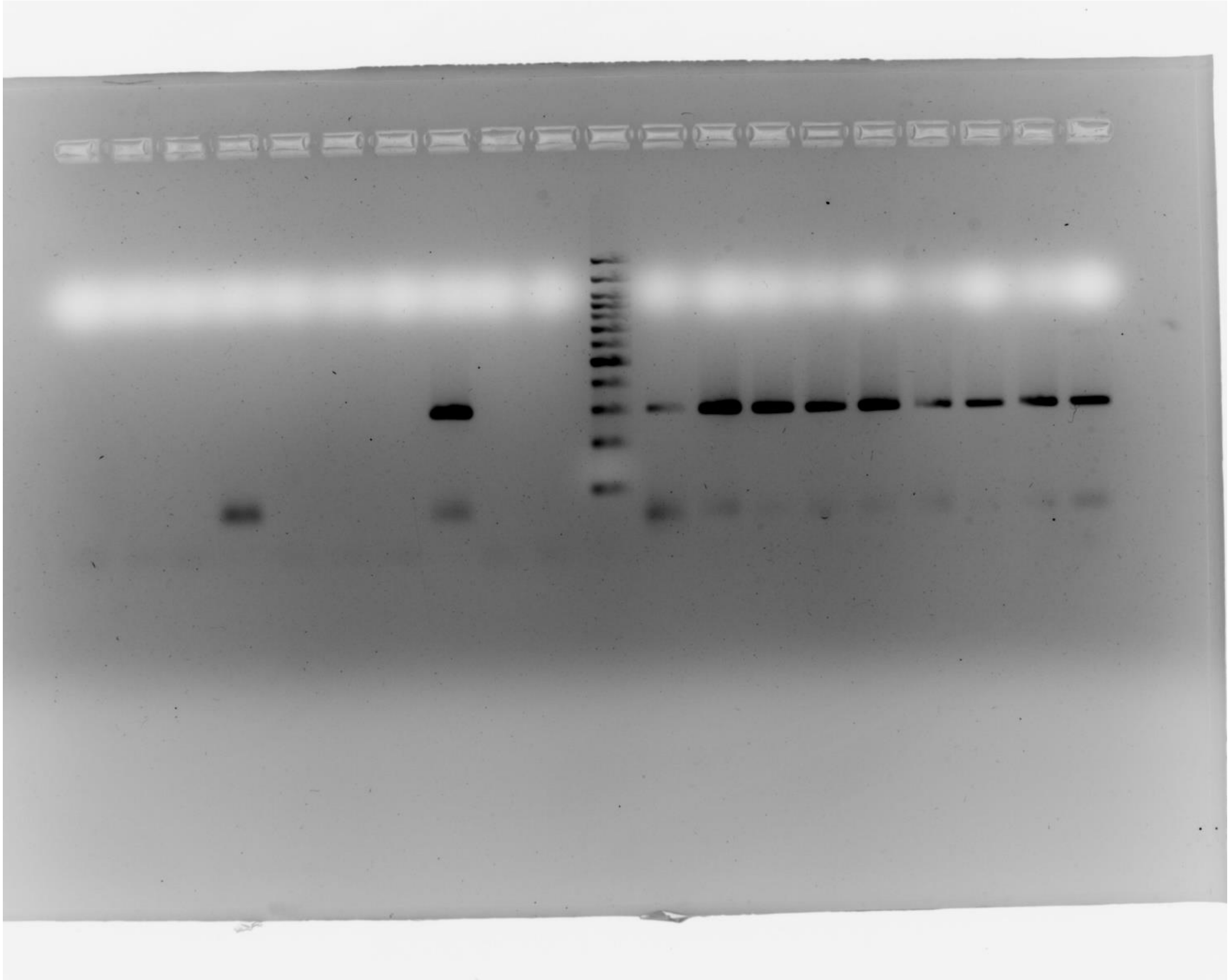

Supplementary Figure S4.

Supplementary References

1. Svanella-Dumas, L. *et al.* Complete nucleotide sequence of a French isolate of Maize rough dwarf virus, a Fijivirus member in the family Reoviridae. *Genome Announc.* **4**, 4–5 (2016).
2. de Haro, L. A. *et al.* Mal de Río Cuarto Virus Infection Triggers the Production of Distinctive Viral-Derived siRNA Profiles in Wheat and Its Planthopper Vector. *Front. Plant Sci.* **8**, 1–11 (2017).
3. Dumón, A. A. D. *et al.* Occurrence of a closely-related isolate to Maize yellow striate virus in wheat plants. *AGRISCIENTIA* **32**, 107–112 (2015).
4. Maurino, F. *et al.* Complete genome sequence of maize yellow striate virus , a new cytorhabdovirus infecting maize and wheat crops in Argentina. *Arch. Virol.* **163**, 291–295 (2018).
5. Truol, G. A. *et al.* Transmisión experimental del virus del Mal de Río Cuarto por Delphacodes kuscheli. *Phytopatologia Bras.* **26**, 39–44 (2001).
6. Sagadín, M. B. & Truol, G. A. Wheat streak mosaic virus (WSMV). in *Enfermedades virales asociadas al cultivo de trigo en Argentina: reconocimiento, importancia, formas de transmisión y manejo.* (ed. Truol, G. A.) 31–40 (INTA, 2009).
7. CICUAE. https://inta.gob.ar/documentos/cicuae-comite-institucional-para-el-cuidado-y-uso-de-animales-de-experimentacion.
8. Percie du Sert, N. *et al.* The ARRIVE guidelines 2.0: Updated guidelines for reporting animal research. *PLOS Biol.* **18**, e3000410 (2020).
9. Vincke, C. *et al.* Generation of Single Domain Antibody Fragments Derived from Camelids and Generation of Manifold Constructs. in *Advances in the Astronautical Sciences* vol. 154 145–176 (2012).
